# Numerical simulation of atmospheric CO_2_ concentration and flux over the Korean Peninsula using WRF-VPRM model during Korus-AQ 2016 campaign

**DOI:** 10.1371/journal.pone.0228106

**Published:** 2020-01-24

**Authors:** Changhyoun Park, Soon-Young Park, Kevin R. Gurney, Christoph Gerbig, Joshua P. DiGangi, Yonghoon Choi, Hwa Woon Lee

**Affiliations:** 1 Institute of Environmental Studies, Pusan National University, Busan, South Korea; 2 Department of Atmospheric Environmental Sciences, Pusan National University, Busan, South Korea; 3 School of Life Sciences, Arizona State University, Arizona, United States of America; 4 Department Biogeochemical Systems, Max Plank Institute for Biogeochemistry, Jena, Germany; 5 National Aeronautics and Space Langley Research Center, Hampton, Virginia, United States of America; University of California Irvine, UNITED STATES

## Abstract

We conducted regional scale CO_2_ simulations using the Weather Research and Forecasting model (WRF) coupled with the Vegetation Photosynthesis and Respiration Model (VPRM). We contrasted simulated concentrations with column, ground and aircraft observations during the Korea-United States Air Quality (KORUS-AQ) 2016 field campaign. Overall, WRF-VPRM slightly underestimates CO_2_ concentrations at ground and column monitoring sites, but it significantly underestimates at an inland tower measurement site, especially within the stable (nocturnal) boundary layer in nighttime. The model successfully captures the airborne vertical profiles but showed a large offset within the planetary boundary layer (PBL) in the areas surrounding Seoul and around the Taeahn point source emissions in the west coastal area of the Korean Peninsula. A case study flight intended to capture Chinese influence observed no clear signals of long-range transport of CO_2_, due mainly to the much larger magnitude of background CO_2_ concentrations. The calculated Net Ecosystem Exchange (NEE) with flux measurements at a tower site in the South Korean Peninsula has also been evaluated comparing with CO_2_ flux measurements at a flux tower site, resulting in the underestimation by less than a factor of 1.

## Introduction

More than half of the global population resides in urban areas, and by 2050, the urban population is expected to increase up to ~ 69% of the world population [1]. As urbanization expands globally, the emissions of anthropogenic greenhouse gases (GHGs) have also increased. About 30%-40% of GHGs are emitted from urban areas, and these anthropogenic emissions are related to severe weather phenomena, such as the increase of average atmospheric temperature, the increase and/or decrease of precipitation, the higher occurrence, and intensity of serious weather phenomena [2]. Despite its low global warming potential relative to other GHGs, the relative abundance of carbon dioxide (CO_2_) emissions make understanding its emission sources critical [3].

A wide variety of studies have been conducted to better understand the effects of CO_2_ on the carbon cycle between the atmosphere and the Earth’s surface, generally using either bottom-up or top-down approaches. A bottom-up approach involves processing information from surveys from individual base anthropogenic inventories, specifying industrial, residential, commercial and transport sectors. These individual inventories are then linked with carbon contents of each fuel type to produce a large, final emission inventory. Errors in this approach are about ±5% uncertainty at the global scale [4] and about 50–200% at the urban scale [5–6]. Upscaling of relations between biospheric CO_2_ flux measurements and environmental factors, such as air temperature, radiation, water, and vegetation parameter, also belong to this approach [7,8]. In contrast, a top-down approach is an empirical downscaling method. For example, in inverse modeling, a predictive atmospheric transport model and *a priori* initial estimates of surface CO_2_ fluxes are used to forecast observed concentrations and to estimate the terrestrial CO_2_ exchange while maintaining consistency between observed and simulated CO_2_ concentrations. The accuracy of this approach is limited by *a priori* initial estimates of CO_2_ fluxes [9, 10].

For decades, neighborhood-scale (microscale) flux measurement studies have been carried out to understand CO_2_ interactions between the surface and the atmosphere. AmeriFlux and FluxNet have played an important role to quantify the Net Ecosystem Exchange (NEE) of CO_2_ [11] in primarily biogenic regions, while efforts to conduct flux measurements in urban areas have been complicated by both anthropogenic emission sources and vegetative uptake [12–14]. Observed concentrations are also used to retrieve NEE via large (global) scale inverse modeling [15–17], However the lack of spatially explicit *a priori* flux estimates (i.e. representative errors) can cause large uncertainties for the production of posterior gridded emission data. Between large scale models and flux measurements, the coarse resolution of global inverse modeling makes it challenging to capture the fine resolution of neighborhood flux measurements. To fill the significant scale-gap between the microscale flux measurements and global inverse modeling, finer spatiotemporal resolution modeling is required.

Mesoscale (regional) meteorological models can be used to establish the link across this scale gap. To this end, a vegetation photosynthesis and respiration model (VPRM) has been coupled in the Weather Research and Forecast (WRF) model [18] and has been built-in to WRF-Chem (hereafter WRF-VPRM) since version 3.4. This coupled model has been evaluated over various vegetation dominated areas, such as croplands and forests and complex mountainous terrain [19–21]. These studies reported significant improvements in capturing the spatiotemporal variation of observed CO_2_ concentrations and fluxes not exhibited specifically in global models. Additionally, the coupled model has been evaluated in urban areas where CO_2_ measurements are complicated by both man-made emission sources and vegetative contributions. Over the southern California region in the US, a sensitivity study focused on fossil fuel CO_2_ emissions revealed that higher resolution input emissions resulted in a better improvement of CO_2_ concentration simulation [22]. Another study over the same domain reported significant improvements in NEE simulation after optimization of VPRM biospheric parameters [23].

The rapid rate of development, and the associated increase in carbon emissions, over the last five decades in East Asia, including China, Taiwan, Japan, and Korea, make it an important target for understanding this coupling between global models and local measurements. Based on the Global Carbon Atlas database [24], the East Asia area is identified as the biggest CO_2_ emission source: China ranked the 1^st^ largest emission country by 26.3% of the world total CO_2_ emissions in 2016, followed by Japan (3.6%), South- and North-Korea (1.9%), and Taiwan (0.8%). Among fossil-fuel territories, coal (68.8%) was the most dominant emission source, followed by oil (17.0%), cement (8.9%) and gas (5.1%) [25,26]. The transportation and industrial processes primarily using fossil-fuel combustion become dominant anthropogenic CO_2_ emission sources. The land-use changes also affect the CO_2_ emissions via the interactions between the surface and the atmosphere: the East Asia uptake about-552 MtCO_2_, which is about 20% of the global emissions 2,488 MtCO_2_ by land-use changes in 2010 [27,28].

In addition to their own domestic emissions, there are also diplomatic controversies among the countries in East Asia, because air pollutants can affect other countries regionally. Specifically, air pollutants emitted from China can move over the West Sea and have been shown to affect the air quality of other nations downwind, such as Taiwan, Japan, and Korea [29–31]. To investigate the factors contributing to poor air quality in Korea, the Korea-United States Air Quality Study (KORUS-AQ) campaign was executed by both Korea’s National Institute of Environmental Research (NIER) and the United States National Aeronautics and Space Administration (NASA) in 2016 spring. The KORUS-AQ study conducted extensive observations of trace gases and aerosols in order to identify specific emission sources, and detailed information can be found at the KORUS-AQ white paper [32].

Among a wide variety of trace gases, in which most of the air pollutants are involved, we focused on CO_2_ in this study. We calculated WRF-VPRM over the Korean Peninsula during the KORUS-AQ 2016 campaign period and evaluated the modeling performance comparing with the concentration and flux observations as well as the meteorology.

## Materials and methods

### WRF-VPRM model

**Fig 1:**
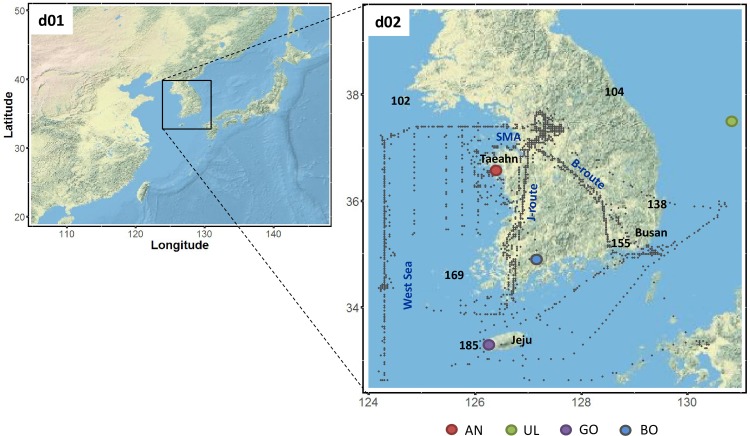
Domains for WRF-VPRM modeling framework. In the fine domain (d02), the DC-8 aircraft 6-min flight tracks marked as dots, and the ground CO_2_ measurement sites are marked with color circles. Three-digit numbers indicate KMA’s rawinsonde sites, and names in blue indicate the flight group for the vertical profile.

In this study, we used VPRM coupled in WRF-Chem (version 3.8.1); more detail can be found in [18]. We utilized two-way nesting with grids at 10 km and 3.3 km resolution (Fig 1) and 38 vertical layers (with 12 layers below 1.5 km) extending up to 100 hPa. Initial conditions (ICs) and boundary conditions (BCs) for meteorological fields for the WRF modeling were fed from the 3-hr GDAS/FNL 0.25 degree global tropospheric analyses and forecast grids data of National Centers for Environmental Prediction [33] and the 6-hr National Centers for Environmental Prediction (NCEP) SST dataset with 8-km horizontal resolution data [34]. The WRF Single Moment (WSM) 3-class microphysics scheme, New-Grell cumulus scheme (only for a coarse domain), and Rapid Radiative Transfer Model (RRTMG) short- and long-wave radiation scheme were used. The Yonsei University scheme [35] with the topo-wind and pbl-mix options were used for the Planetary Boundary Layer (PBL), which we determined based on sensitivity tests (S1 Table). The WRF four-dimensional data assimilation (FDDA) was applied using the grid nudging options. We conducted the numerical simulation during two periods: (1) from April 26 00:00 UTC to May 11 00:00 UTC, and (2) from May 12 00:00 UTC to June 10 00:00 UTC, with 5 days spin-up time for each simulation.

### Vegetation photosynthesis and respiration model

Biospheric CO_2_ fluxes were calculated in the VPRM module, using land-use and satellite data, and simulated air temperature and radiation [18]. In detail, vegetation is classified into 8 land-use categories, including evergreen forest, deciduous broadleaf forest, mixed forest, shrubs, savanna, cropland, grassland, and urban/water area, based on the 1-km global Synergetic Land Cover Product (SYNMAP) data [36]. Each vegetation category has its own biospheric parameters, called VRPM parameters.

The Enhanced Vegetation Index (EVI) and Land Surface Water Index (LSWI) from the Moderate Resolution Imaging Spectroradiometer (MODIS) surface reflectance data are used to generate the scaled temperature (T_scale_), physiological index (P_scale_), and canopy water content (W_scale_). These scaling factors and the VPRM parameters, including the maximum quantum yield (λ) and the half-saturation value of photosynthetically active radiation (PAR_0_), are used to calculate the Gross Ecosystem Exchange (GEE). The simulated shortwave radiation (SW) is used for the photosynthetically active radiation (PAR), as SW is strongly correlated with PAR: SW ≈ 0.505 × PAR (units: SW, W m^-2^; PAR, μmol m^-2^ s^-1^) [18]. The respiration rate (RESP) is calculated from the WRF’s 2-m air temperature (T) along with first order linear parameters, including a slope (α), and an intercept (β). The summarized equations for the calculation of NEE (GEE + RESP) used in the VPRM module are below:

$$GEE=\lambda \times T_{scale}\times W_{scale}\times P_{scale}\times \frac{1}{\left(1+PAR/{PAR}_{0}\right)}\times PAR\times EVI$$ (1)

RESP=α×T+β (2)

The VPRM parameters should be optimized based on local CO_2_ flux measurements for each representative land-use class. However, due to the lack of observations over the study domain during the study period, we used the default VPRM parameters.

### Fossil fuel CO_2_ emissions

In this study, we used the NOAA 2016 CarbonTracker 3D CO_2_ mole fraction data (version CT2017 for CO_2_ lateral boundary and initial conditions. The 3-hr CT2017 data, containing global background, photosynthesis/respiration by biosphere, fires, combustion of fossil fuels, and air-sea exchange, covers all globe up to the tropopause, with 2.5° × 2.5° spatial resolution [37]. To feed surface boundary conditions for CO_2_, hourly fossil fuel CO_2_ emission data for the year 2015 from the Fossil-Fuel Data Assimilation System (FFDAS) [38], gridded at 10 km, were used. The FFDAS is a data assimilation system that estimates the fossil fuel CO_2_ emissions at every grid cell by solving the Kaya identity, which expresses emissions as a product of population density, per capita economic activity, energy intensity of the economy, and carbon intensity of energy [39]. A series of spatially explicit observation data are used to constrain each factor in the Kaya identity. The FFDAS uses remote sensing-based nighttime lights data, gridded population sector-based fossil fuel CO_2_ emissions from the International Energy Agency (IEA), and global power plant CO_2_ emissions [6]. Natively, the FFDAS estimates fossil fuel CO_2_ emissions at 0.1° and annual resolutions over the globe. Nasaar et al. [40] derived scale factors that can apply to global emission data to represent the diurnal and weekly cycles of fossil fuel CO_2_ emission variation at 0.25° × 0.25° resolution, which we used for this study.

### Observation data

In order to better understand the factors contributing to poor air quality in Korea, the KORUS-AQ campaign was performed by both Korea’s National Institute of Environmental Research (NIER) and the United States National Aeronautics and Space Administration (NASA) from May to early June 2016. The KORUS-AQ study conducted extensively spatial and vertical observations of both trace gases and aerosols using ground sites, aircraft, and vessels in order to identify specific emission sources and support policymakers to establish air quality mitigation strategies. Detailed information is available on NASA’s KORUS-AQ webpage [41].

The NASA DC-8 aircraft was instrumented to measure vertical profiles of meteorological variables and chemical species. Ambient CO_2_ dry molar ratios were measured using a modified commercial non-dispersive infrared (NDIR) spectrometer (LI-COR 6252) with a combined precision and accuracy of 0.1 ppm (1σ) at a 1 Hz sampling rate. In addition, we used the 92 Automated Synoptic Observation System (ASOS) data and 6 rawinsonde data of the Korean Meteorological Administration (KMA) to evaluate the meteorological modeling performance. In order to estimate simulated column concentrations, we used the Total Carbon Column Observing Network (TCCON) data [42].

We also evaluated ground level CO_2_ concentrations using a variety of measurements at different sites, summarized in Table 1. These include the World Meteorological Organization Global Atmosphere Watch (WMO GAW) data [43] at not only Anmyeondo (AN), Jeju Gosan (GO) and Uleungdo (UL) but also Boseong (BO). BO is a tall tower site which can measure vertical profiles of CO_2_ at 60 m, 140 m and 300 m a.g.l., but only 60-m and 300-m data were available during the study period. The instrumentation is located in the middle of cropland, mainly consisting of rice paddies, that opens to a bay area to the south and is surrounded by hills in the other directions ~ 2–3 km from the tower. Small residential houses and farming warehouses are located at the mountain base.

**Table 1:** Description of ground and column CO_2_ measurements.

Site	Latitude, Longitude	Elevation (m a.g.l)	Contributor	CO_2_ Instrument
**Ground concentration observation**				
**UL**	37°28'48"N, 130°54'00"E	10	KMA	G2301 (Picarro, USA)
**AN**	36°32'19"N, 126°19'48"E	40	WMO GAW of KMA (AMY)	G2301 (Picarro, USA)
**GO**	33°10'48"N, 126° 7'12"E	12	WMO GAW of KMA (JGS)	G1301 (Picarro, USA)
**BO**	34°45’47.95”N, 127°12’49.31”E	60, 300	KMA	Li-7500 (Licor, USA)
**Column concentration observation**				
**AN**	36°32'19"N, 126°19'48"E	25	WMO GAW of KMA (AMY)	IFS-125HR (Bruker, Germany)

The Picarro ground instruments were calibrated weekly.

## Results and discussion

The simulated results for the finest domain (d02) have been evaluated by comparing with column, ground, aircraft and flux observations from May 1 to June 10, 2016. The basic statistical measures used mainly here are the Mean Bias (MB), the Root Mean Square Error (RMSE), and the Index of Agreement (IOA [44]) The individual equations can be expressed as below:

$$RMSE=\sqrt{\sum_{i=1}^{N}\frac{{\left(M_{i}-O_{i}\right)}^{2}}{N}}$$ (3)

$$MB=\frac{\sum_{i=1}^{N}\left(M_{i}-O_{i}\right)}{N}$$ (4)

$$IOA=1-\frac{\sum_{i=1}^{N}{\left(M_{i}-O_{i}\right)}^{2}}{\sum_{i=1}^{N}{\left(\left|M_{i}-\frac{\sum_{i=1}^{N}O_{i}}{N}|+|O_{i}-\frac{\sum_{i=1}^{N}M_{i}}{N}\right|\right)}^{2}},$$ (5)

where, *M_i_* and *O_i_* indicate modeling results and observations, respectively, at each grid point (*i*). The time used in this manuscript is South Korean Local Standard Time (LST) unless otherwise specified.

### Meteorological evaluation

We compared the simulated meteorological results from WRF-VPRM with 93 ground measurements at ASOS sites run by KMA. The observed, averaged 2-m temperature, which is an important meteorological factor for the calculation of vegetation respiration in the VPRM module, was 18.5°C with the model underestimating by 1.0°C (IOA = 0.94). The averaged 10-m wind speed was 2.1 m s^-1^ with the model underestimating by 0.2 m s^-1^ (IOA = 0.81). The 10-m wind directions ranged from 190–280°, and the dominant wind direction was SW and W during the study period, except May 10 and 11 (NE wind direction). The simulated offset in wind direction was < 15°in average.

The vertical profiles of simulated meteorology were also evaluated with 6 rawinsonde observations [45]. The model underestimated the potential temperature by 0.1°C and wind speeds by 0.4 m s^-1^. The simulated PBL height (PBLH) is also critical, because trace gas concentrations are ultimately determined by the volume of PBL. PBLH was calculated using the bulk Richardson number [46]. WRF-VPRM successfully captured the diurnal variation in PBLH, which began to increase at ~ 06:00 LST, reached a peak at ~ 15:00 LST, and then decreased until ~ 20:00 LST. The model underestimated by ~ 73 m in average.

### Comparison with ground observations

#### Horizontal spatiotemporal distribution

**Fig 2:**
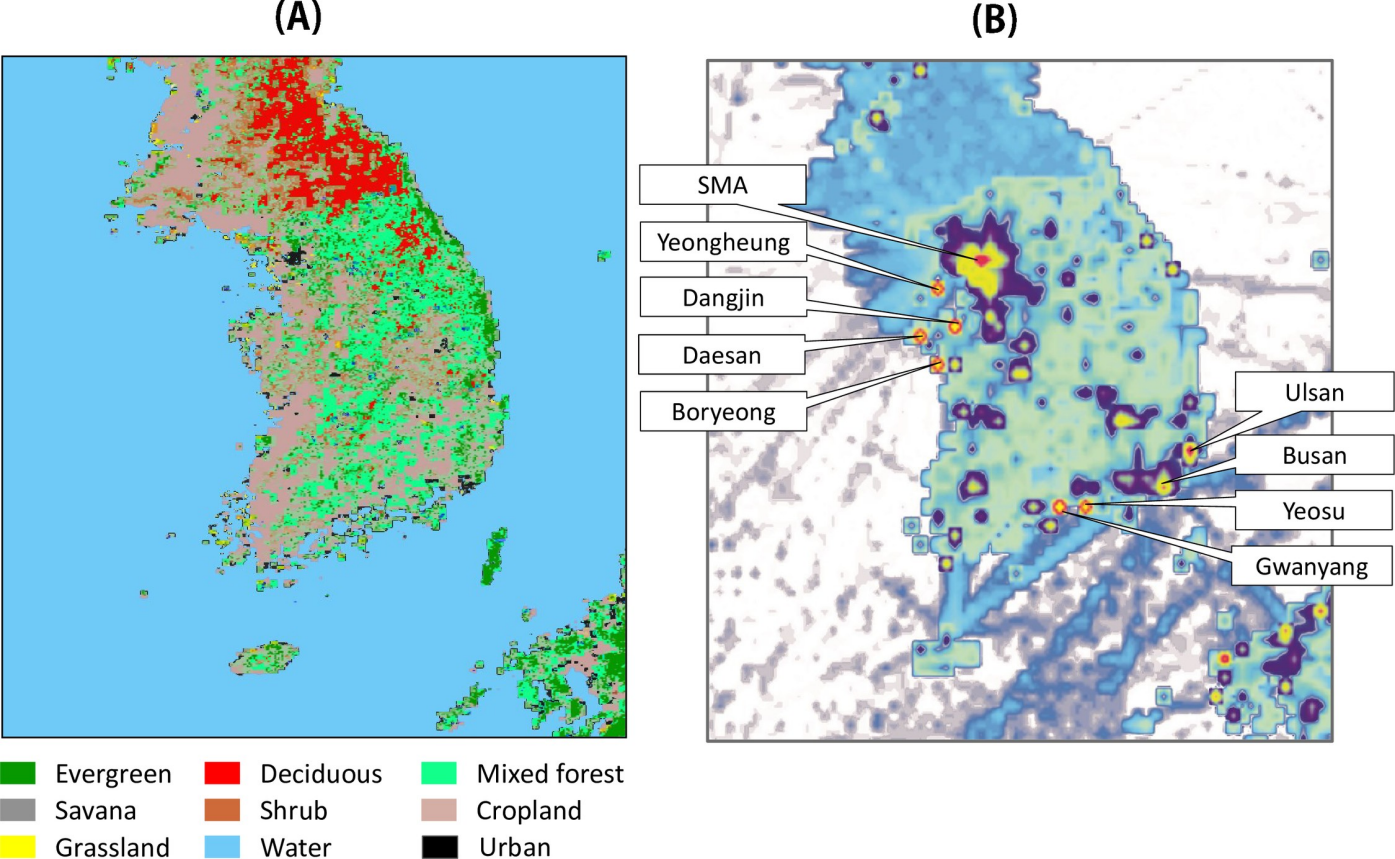
**Spatial distribution of (a) land cover retrieved from SYNMAP and (b) anthropogenic emission from FDDA emission data in the finest domain.** The ranges of anthropogenic emissions are displayed in S1 Fig. Names in square boxes indicate top 9 highest emission areas in Korea.

Spatiotemporal variations in atmospheric CO_2_ concentrations are typically associated with the spatial distribution of emission sources and biosphere combined with the temporal variations in PBLH. Fig 2 shows the horizontal distribution of land cover retrieved from SYNMAP and emissions from FFDAS in the study domain. The significant point sources are mainly located in the west side of mid-Korea, mostly in and near the Taeahn peninsula, in which Yeongheung and Boryeong power plants and Daesan and Dangjin industrial complexes exist. Gwangyang and Yeosu industrial areas are also identified as large point sources in the south. Among several major cities of Korea, Seoul Metropolitan Area and its vicinity (SMA, a capital area) showed the largest area emissions, followed by Ulsan (Korean largest petrochemical industrial city), and Busan (Korean largest harbor city). The ranges of emissions are reported by color code in S1 Fig.

The highest CO_2_ concentrations appeared in the early morning (06:00–09:00 LST), associated with the lowest PBLH and the morning rush-hour vehicle emissions (not shown). Around noon, when the PBL fully developed and vegetation uptake thoroughly increased, the CO_2_ concentration remained low until late afternoon. After that, the concentration began to gradually increase again by the start of evening rush-hour while PBLH decreased. The increased concentration remained high through the night until early next morning.

#### Column and ground concentrations

Before the further comparison of the simulated results with the ground and vertical observations, it is necessary to confirm the total mass consistency in the modeling domain. A total column concentration of CO_2_ simulated can be compared with the observed column CO_2_ concentration (xCO_2_) data acquired from the TCCON site at AN. The averaged xCO_2_ showed 405.4±0.7 ppm, and the vertical accumulated concentration of WRF-VPRM was underestimated by approximately 0.3 ppm, which is comparable to the resolution of TCCON (0.25 ppm).

At the Korean CO_2_ background monitoring sites at AN, GO and UL observed mean concentrations of 414.1±2.9 ppm, 411.9±4.4 ppm, and 410.5±3.9 ppm, respectively, during the study period. Assuming that the UL mean is the background concentration of our study domain (Korea), it was about 2–7 ppm higher than the global CO_2_ background measured at Mauna Loa (408±1 ppm) in May 2016 [47]. At each CO_2_ monitoring site, WRF-VPRM underestimated the CO_2_ concentration at AN by 2.4 ppm (RMSE = 3.6) and at GO by 0.4 ppm (RMSE = 4.7) and overestimated at UL by 0.7 ppm (RMSE = 4.6), on average. The larger MB at AN is likely from the relatively more sparse observations (only four days) compared with the other two sites.

Besides the uncertainties from simulated meteorology, the impact of the temporal scaling factors in the FFDAS emission data, which enables annual global emission data to have a diurnal and weekly cycle, can introduce uncertainty in our simulations. Based on sensitivity tests using GEOS-Chem [40], the impact of the scaling factor on the surface atmospheric CO_2_ was negligible for areas far from sources and ranged approximately from 1.5 to 8 ppm over large urban areas. Its effect on the xCO_2_ was as high as 0.1–0.5 ppm over the major urban areas. Judging on the locations of the three CO_2_ monitoring sites, which are set up among the areas far-from and nearby sources, our simulation MB is tolerable.

**Fig 3:**
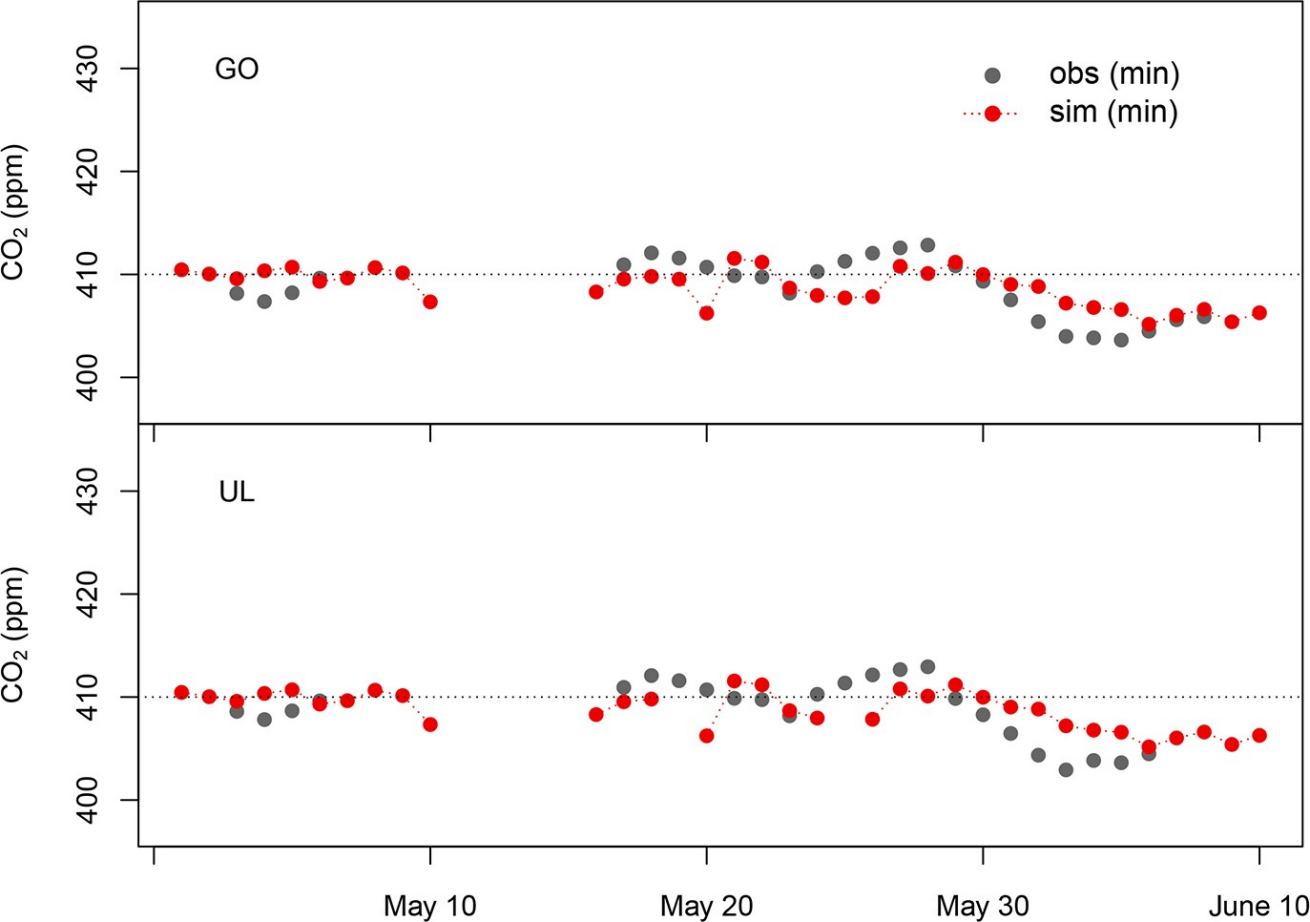
5-day moving average of observed and simulated daily minimum CO_2_ concentrations at GO and UL.

The model captured the clear diurnal variations: dominant peaks appeared at 06:00–07:00 LST due to the CO_2_ emitted on the early morning and/or carried-over from the previous day when PBLHs were still low. In spring, as the vegetation starts to grow, it is expected that the CO_2_ concentration will gradually decrease as biospheric uptake increases. The variation of daily minimum CO_2_ observations, which can minimize the influence of erratic anthropogenic CO_2_, can be a good tool to check the temporal trend. Fig 3 shows the 5-day moving average of observed and simulated daily minimum CO_2_ concentrations, indicating that our model successfully captured the CO_2_ decreasing trend as summer comes.

**Fig 4:**
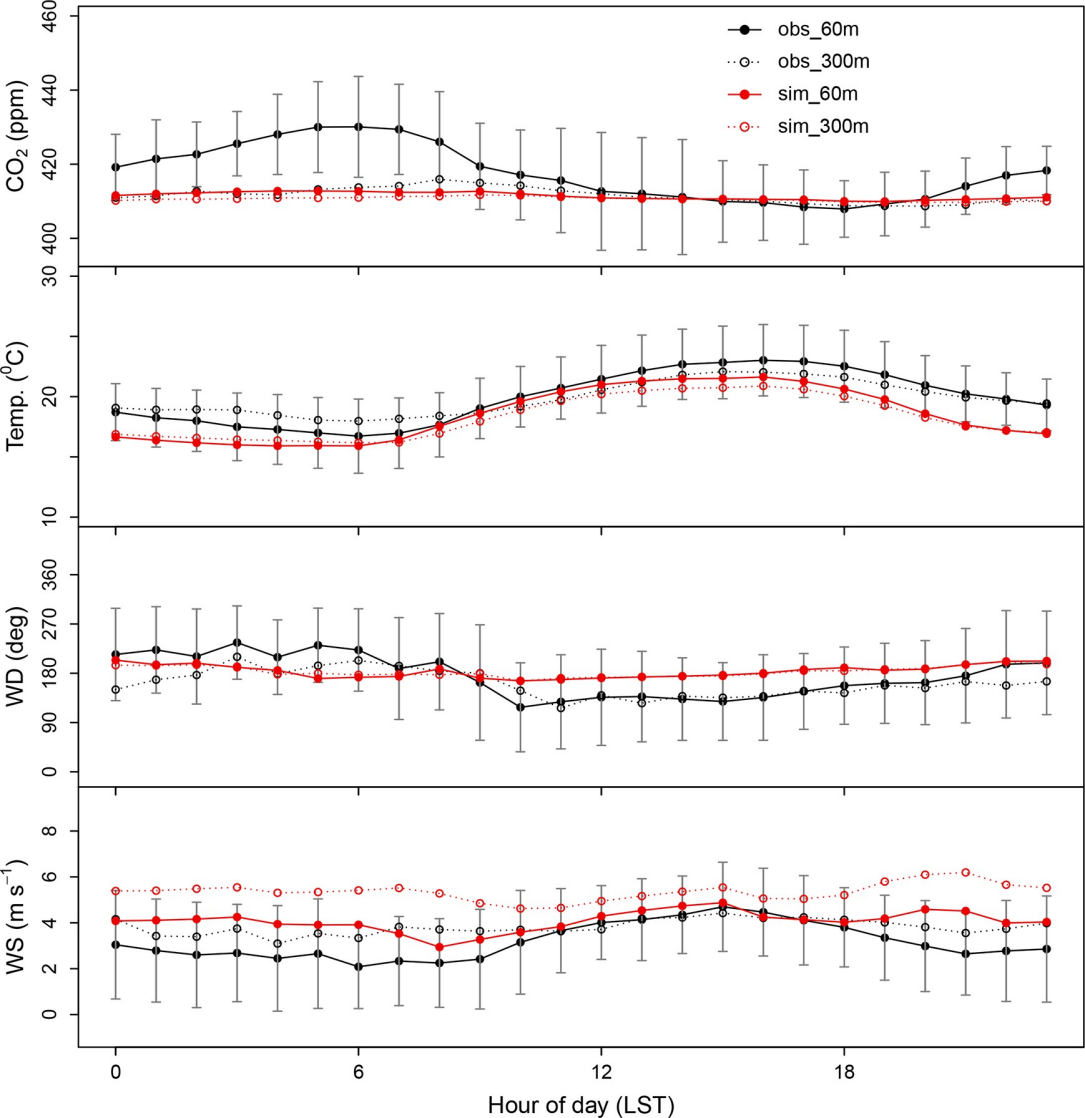
Diurnal variation of CO_2_ concentrations and meteorology, observed and simulated, at the flux tower site (BO) at 60 m and 300 m a.g.l. Vertical gray lines indicate 1- σ of observations.

In addition to evaluation using ground CO_2_ monitoring sites, we estimated the modeling performance with a tall tower observation at BO (Fig 1) at both 60 m and 300 m a.g.l. Note that vertical CO_2_ concentrations provided by tower sites were only available at BO during the study period. Fig 4 shows the averaged diurnal variation of the observed and simulated CO_2_ concentrations, temperature and winds at each level. The observations at 60 m and 300 m showed a peak at ~06:00 LST and ~09:00 LST, respectively, then both concentrations gradually decreased until ~ 18:00 LST and then increased through the night. The concentrations at the lower level showed 3.8 ppm and 9.7 ppm higher than that at the upper level in day- and night-time, respectively. The larger difference at night can be interpreted at the point of view of micrometeorology. At night, the 60 m level was within the stable (nocturnal) boundary layer, while the 300 m level was located in the residual layer. The nighttime inversion was observed in temperature as well, and the averaged PBLH calculated by WRF was ~100m under the assumption that we can treat the nighttime PBLH as the top of the stable boundary layer. Interestingly, the inversion appeared unique to the study period, as it was not observed in the other spring (S2 Fig). During mid-day, in contrast, there was much less difference in concentrations (~ 2 ppm) because the two levels were together within the mixed layer. Overall, the observations showed a typical diurnal variation of CO_2_ concentration within PBL.

Our model successfully captured the diurnal variation of CO_2_ concentrations at 300m, and the simulated results underestimated by approximately 3.9 ppm (RMSE = 9.6) and 0.5 ppm (RMSE = 18.8) in day- and nighttime, respectively. At 60 m, on the other hand, the model failed to capture the amplitude of temporal variations and significantly underestimated nighttime concentrations by 10.3 ppm (RMSE = 14.3). The uncertainties of WRF-VPRM can be caused by vertical mixing, advection, initial fields, emissions and VPRM parameters, described in previous WRF-VPRM studies [19,21]. Considering the model’s reliable simulation performance in micrometeorology, as previously described, the significant offset of CO_2_ concentration at 60 m was likely due to the uncertainty of emissions around BO. The prevailing winds were from the south, where the local emission sources, such as fishery boats, vehicles, small factories and residential houses in the bay area, are located. These small emission sources are not mandatory to report, so they are not likely fully represented in the relatively coarser resolution of emission inventories. In sum, due to the underestimated nighttime emissions at each grid in model, the model failed to capture the accumulated concentrations within the stable (nocturnal) boundary layer, at least around BO during the study period.

We also evaluated the vertical gradient in CO_2_ concentrations, using the daily minimum values at two levels, resulting in that the model underestimated by about a factor of 2, due to the significant underestimation of CO_2_ concentration as described above. It is not clear whether this relatively poorer performance can be also shown in other areas, due to the sparser tower observations. Instead, aircraft observations are helpful for estimating the vertical gradient at higher levels.

### Comparison with aircraft measurements

In this section, we evaluated WRF-VPRM’s performance with the NASA DC-8 aircraft observations. The 4-D fields of simulated CO_2_ concentrations were collocated with flight tracks in space and time at each hourly simulation output. The averaged offset of pressure between the simulated results and the DC-8 observations was ~10±15 hPa in total 18-flight days. In each day of observations, DC-8 took off and flew along planned routes (Fig 1). For the clarity, we divided the aircraft observations into four groups, based on the land cover below the flight tracks and the type of emission sources: SMA, the West (Yellow) Sea, and two main domestic airways in Korea. The SMA represents air quality over the Korean capital city, in which anthropogenic combustion emissions are dominant. The flight tracks over the West (Yellow) Sea were intended to detect the long-range transport of air pollutants from China, where the DC-8 took meridional tracks over the ocean forming a “wall” between China and the Korean Peninsula. The two domestic airways, connecting Seoul to Jeju (J-route) and Busan (B-route), which are the most frequent observation routes that DC-8 took during the study period. The J-route was designed to sample the air over local point sources, transported air from the West Sea, and croplands. In contrast, the B-route was designed to capture the biospheric activity mostly over mountains and the Busan urban emission.

**Fig 5:**
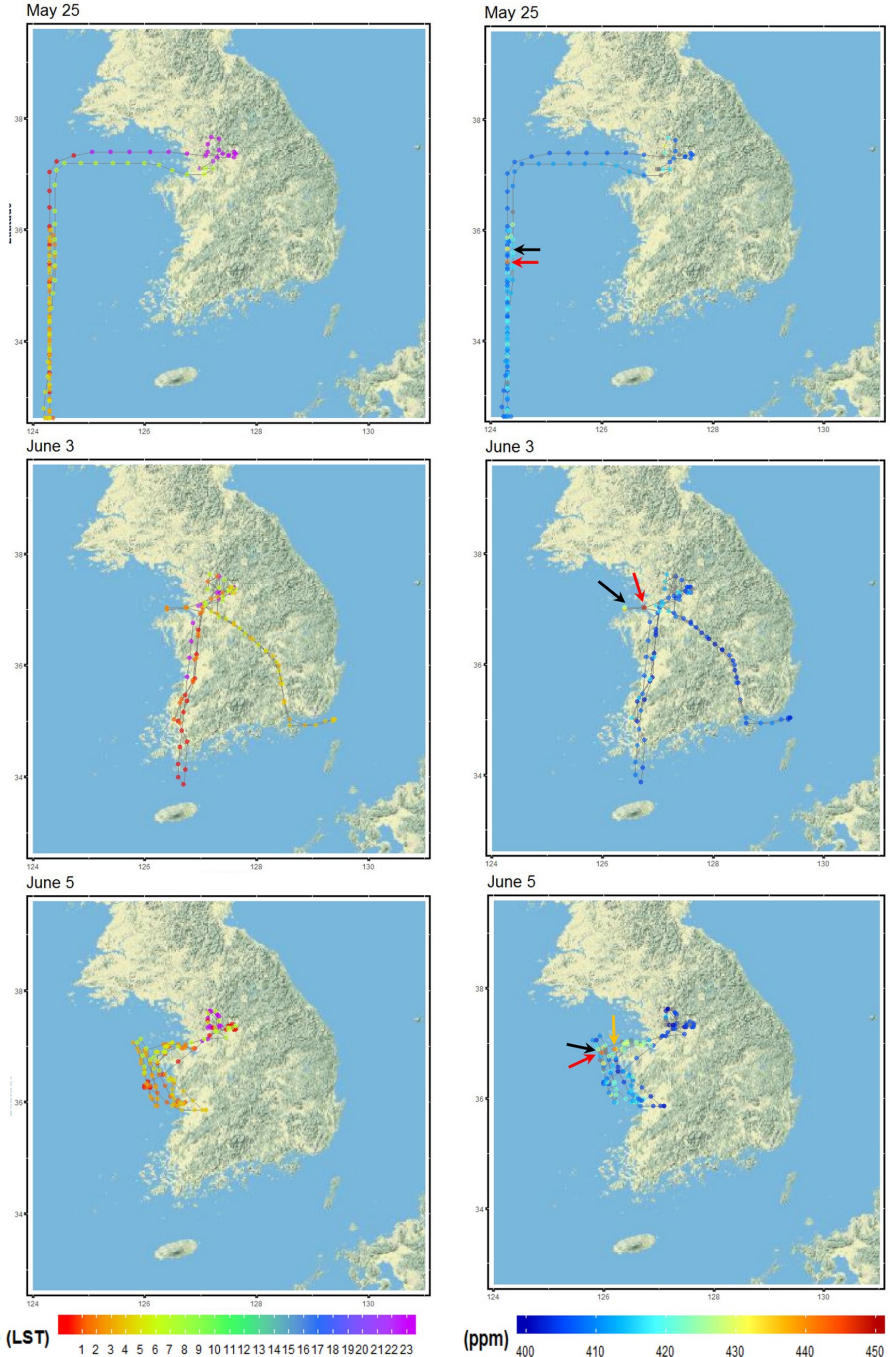
Flight time and CO_2_ concentrations observed along the DC-8 flight tracks on May 25, and June 3 and 5. The colored arrows indicate the peaks marked in Fig 6.

**Fig 6:**
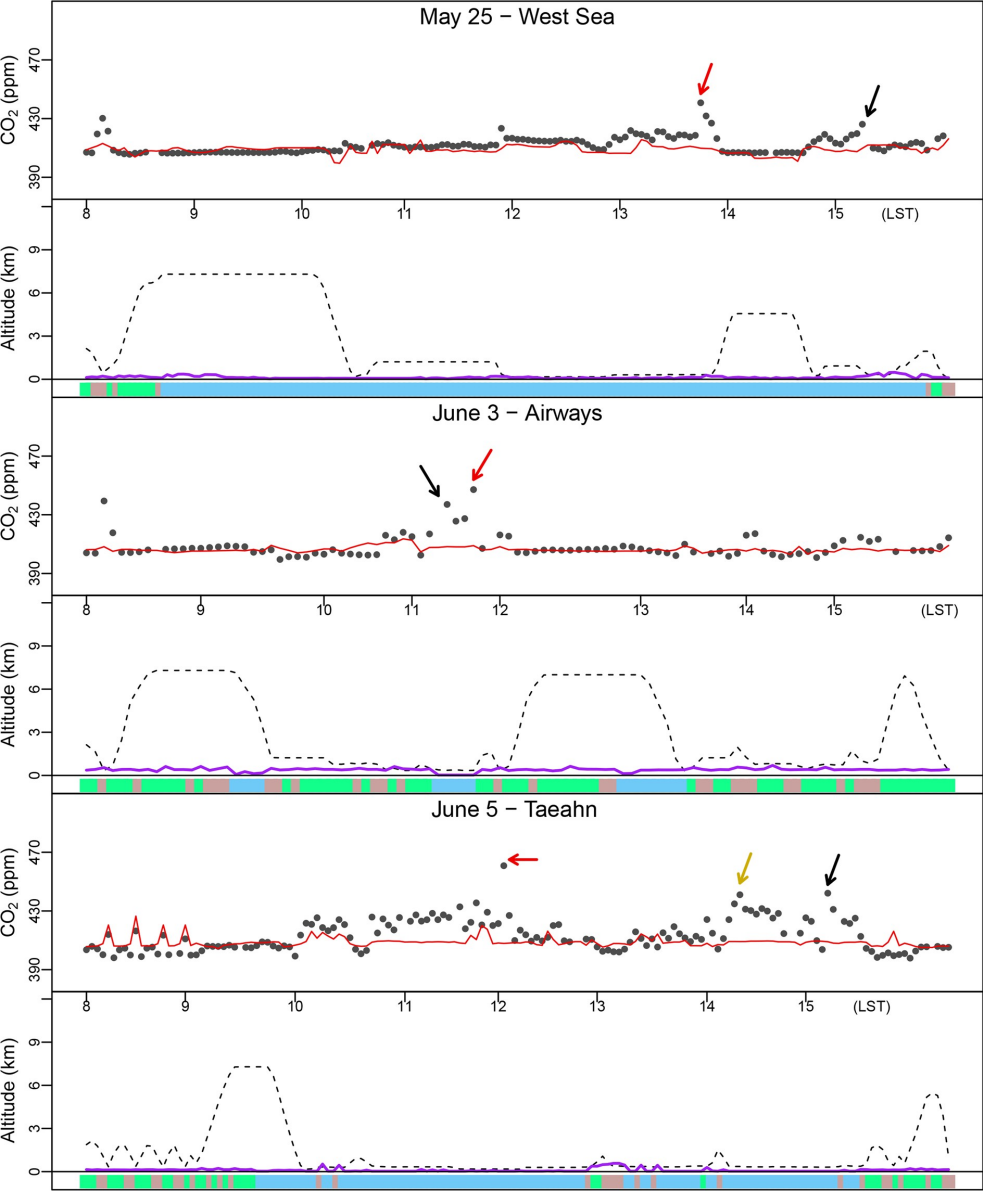
**Time series of** CO_2_ **concentrations,** simulated (red solid lines) and observed (black dots), in each upper panel; and DC-8 flight altitude (black dashed line) and simulated PBLH (purple solid line) over the land use with color code in each bottom panel. Land-cover types at the aircraft locations are represented by color codes as Fig 1. The colored arrows indicate the dominant peaks and their flight locations are marked in Fig 5.

**Fig 7:**
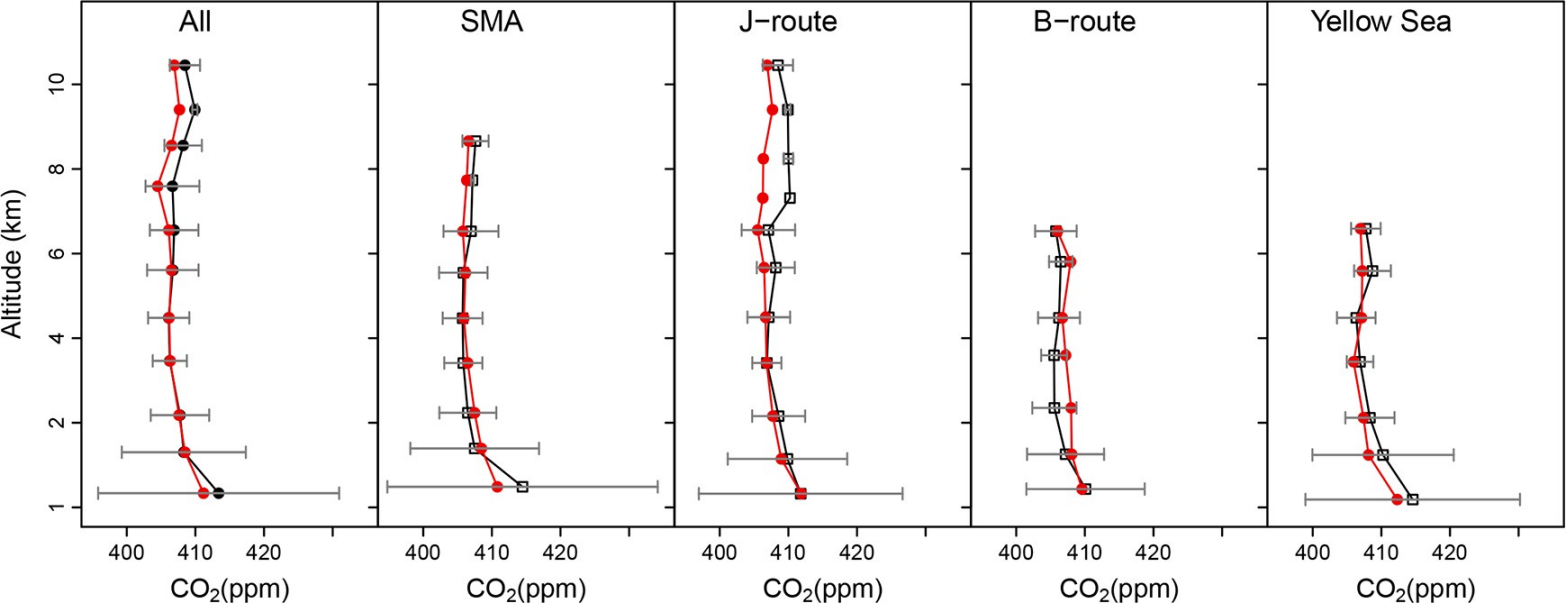
Vertical profile of CO_2_ concentrations for each section, including SMA, J- and B-route and the West Sea as well as all flights during the study period. Red and black dot-lines indicate WRF-VPRM results and NASA DC-8 aircraft observations, respectively.

Fig 5 displays DC-8 flight tracks with observed CO_2_ concentrations on representative flight days: May 25 mainly over the West Sea, June 3 for the domestic airways, and June 5 near the Taeahn peninsula. We evaluated the model’s performance based on the analysis of time series and vertical profiles of each group (Fig 6 and Fig 7). The full set of time series for 18 individual flights is provided in S3 Fig and S4 Fig. During the study period, the aircraft flew over various land-cover and land-use types, maneuvering in and out of the PBL. Overall, as expected, the lower concentrations appeared at higher altitudes and/or over the ocean, while the higher concentrations emerged over urban areas and/or within the PBL. In the vertical profiles in Fig 7, observed CO_2_ concentrations exhibited the maximum concentrations at < 1 km and gradually decreased along with the height up to 7–10 km, showing the minima at about 2–3 km. Overall, the averaged concentration observed was 410.2±5.2 ppm across all flights. WRF-VPRM underestimated concentrations by 1.7 ppm (RMSE = 6.9, IOA = 0.6), and the averaged CO_2_ concentrations above the PBL ranged from 407.1 ppm to 409.7 ppm, while those within the PBL showed the largest biases mainly from SMA.

#### The Seoul metropolitan area (SMA)

About 55% of the measurements were taken below ~1.5 km by the DC-8 when it flew over the SMA. Within the PBL, this group showed the highest averaged concentration of 420.6 ppm compared with other groups. This high value is most likely to be driven by Seoul populated local emissions. WRF-VPRM captured the CO_2_ vertical profile over the SMA like other groups, except near the ground (Fig 7). The simulated results underestimate concentrations by 7.3 ppm and 0.4 ppm within and above the PBL, respectively. The large bias in the PBL concentrations may be caused by the simulated deficiency from both/either the vertical gradient CO_2_ near the ground and/or the incompletely established emissions over the SMA. We roughly assumed that the vertical gradient of CO_2_ concentration within the PBL can be calculated by the difference in concentration between the ground and the 2-km level. The model underestimated the vertical gradient by ~ 30%. This implies that densely populated emissions in Seoul are not fully resolved in our model and it causes the underestimation. Tang et al. [48] also reported a similar poor result in the same study domain and period, using a different model, the Copernicus Atmosphere Monitoring Service (CAMS), and pointed out the limit of the model’s resolution.

#### The West (Yellow) Sea

**Fig 8:**
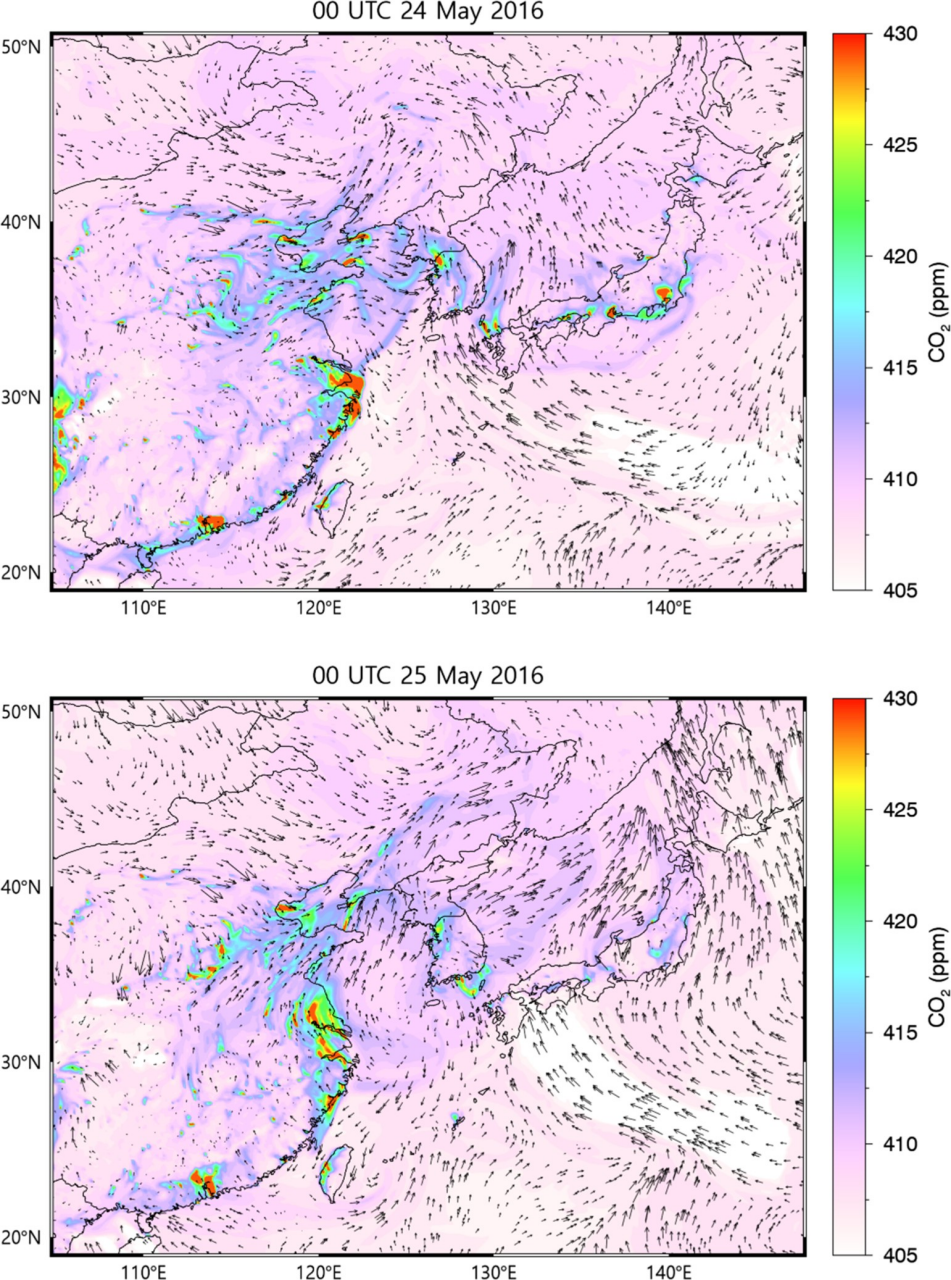
**Spatiotemporal variations of CO_2_ concentrations over the coarse domain at the ground level** on May 24 (upper panel) and 25 (bottom panel) at 09:00 LST.

The effects on CO_2_ concentrations from long-range advection from the outside of the Korean Peninsula should be also considered. Legs of DC-8 flight tracks on May 4, 18, 25, 30, and 31 (LST) were designed to detect outflow plumes from China, flying along the “wall” tracks over the West Sea. We displayed the simulated results over East Asia on May 25, for example, in Fig 8. The polluted air plumes starting from highly populated cities, such as Shanghai, Tianjin, and Hangzhou in China, flew in westerlies and arrived to the “wall” within a day. The averaged observed CO_2_ concentration was 410.9 ppm and was underestimated by WRF-VPRM by ~2.3 ppm (RMSE = 5.5) for the five days of flight tracks along the wall. In order to quantify the magnitude of China’s outflow, we conducted additional numerical experiments in which we used the same simulation configurations, described in Sect. 2, but enabling only China’s FFDAS emissions in order to to detect the simulated advection of CO_2_ originating from China. It was expected that the concentration difference between the two cases under westerly wind conditions would help quantify the effects of long-range CO_2_ transport at the “wall”. However, the averaged difference was ~0.2 ppm (RMSE = 0.6; IOA = 0.99), which is too low to clearly quantify the effects of China’s outflow compared with the atmospheric background CO_2_ concentration. It also agrees to the results of Tang et al. [48].

#### Seoul-Jeju and Seoul-Busan airways

Among several domestic commercial airways in Korea, two routes (J- and B-route) were chosen for the investigation by DC-8 aircraft observations. The J-route is close to the western coastal areas of the Korean Peninsula and has more anthropogenic emission sources and croplands, while the B-route is located over more forested inland areas. In May 2016, the J- and B-route had 8,176 and 1,646 flights in total, respectively, including both passenger and freight flights, based on statistics from Korea Airports Corporation [49]. However, the effects of this number of domestic aircraft on CO_2_ concentrations is minor compared with the surface anthropogenic emissions below the flight tracks. In the South Korean Peninsula, there are a lot of huge point sources, such as fossil-fuel power plants and industrial areas. About 30% of the total 39 fossil-fuel power plants of Korea in 2016 were located in the western part of Korea, and they generated energy of ~ 16 GW out of ~ 32 GW of all Korean fossil-fuel power plants, reported by the electric power statistics information system [50]. Overall, the averaged concentrations on J- and B-route were 410.3 ppm and 408.1 ppm, respectively.

The effects of the point sources on the simulated results were also very apparent. On June 2 and 3 (Fig 5), for example, the flight tracks were designed to cover both J- and B-routes. DC-8 detected hot spots (up to ~ 450 ppm) for two consecutive days, when the aircraft made a slight detour from the direct J-route to the northern tip of the Taeahn peninsula. The uncertainty caused by the point sources can be clearer by removing these hot spots in DC-8 observations. After removing the peaks, the MB in the simulated results was significantly reduced by a factor of ~ 5. On June 5 (Fig 5), the flight track was specifically designed for a more intense survey of point sources around the Taeahn peninsula. Comparing with other aircraft observations, the simulated results failed to capture these huge peaks and significantly underestimated CO_2_ by 4.6 ppm (RMSE = 11.5). The poorer statistics are due mainly to the scattered point sources that are not fully resolved and/or that are blended in coarser grids compared with the spatial scale of aircraft observations. That is, while the aircraft observed air pollutants at the subgrid scale, the model was not able to fully detect them within its lower resolution (3.3 km). Regional scale inverse modeling can help us better estimate the locations of these local sources using aircraft-based prior observations, as shown by Brioude et al. [51]. However, this is outside of the scope of this study.

### CO_2_ fluxes

**Fig 9:**
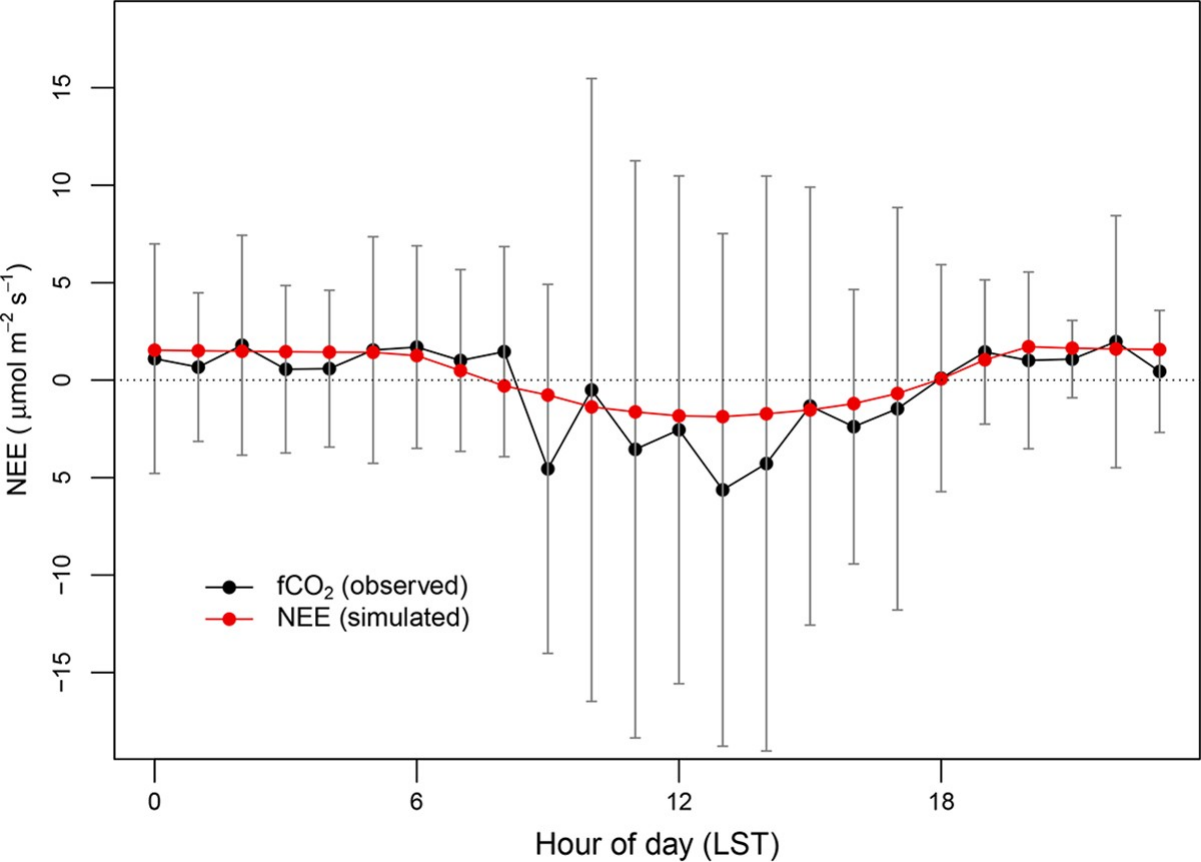
Averaged diurnal variation of simulated NEEs and observed CO_2_ fluxes at BO (60 m). Vertical gray lines indicate 1-σ of observations.

Although CO_2_ is chemically inert in the atmosphere, it continuously interacts with the biosphere, so CO_2_ flux simulation is necessary to estimate atmospheric CO_2_. The interactions can be represented by NEE. VPRM provides the NEE as the sum of GEE and RESP (Sect. 2.2). The simulated NEE can be evaluated by comparing with measured CO_2_ fluxes for each vegetation type. Under the assumption of that the simulated NEE equals to the measured CO_2_ flux as suggested by Park et al. [23], we compared the observed flux data at the ground level with the NEE at BO during the study period (Fig 9). The simulated results showed the clear diurnal effects of vegetation: the daytime negative values due to photosynthesis and the nighttime positive values driven by biospheric respiration. The time series of NEE showed increased intensity in summer, which is inversely correlated with decreasing of CO_2_ concentrations (Sect. 4.2). Median values of observed fluxes were -0.20 μmol m^-2^ s^-1^ and 0.84 μmol m^-2^ s^-1^, and our model showed approximately +98% and -52% offset in day- and night-time, respectively.

The uncertainty in the simulated NEE can be influenced by several factors. First, errors in simulated 2-m air temperature can generate biased NEE, as pointed out in previous studies [18,20]. Our validation statistics showed the simulated 2-m temperature was quite close to observations, so the meteorological uncertainty can likely be neglected. Second, direct comparison of simulated NEEs with measured CO_2_ fluxes can also cause large uncertainties, because the flux measurement naturally includes a CO_2_ storage term within the canopy [52]. The bigger the storage term of CO_2_ fluxes, the larger offset in NEEs. The canopy within the fetch of the flux site at BO dominantly consists of short rice fields, which would minimize the uncertainty from the storage term bias compared with tall and large-bodied tree canopies. Third, VPRM parameters can add uncertainty to the NEE. As described in Sect. 3, the VPRM parameters represent vegetation characteristics, such as light efficiency, photosynthetic active rate, and respiration, which should be optimized based on the flux measurement data. A few studies applying this optimization have been conducted: Jamroensan [20] improved CO_2_ modeling performance by adding a new category of vegetation such as soybean over the Midwestern US; Park et al. [23] also improved the NEE simulation results, optimizing VPRM parameters over the Southern California basin.

The VPRM parameters should ideally be optimized at flux measurement sites in various vegetation types. We could not conduct this optimization due to the lack of flux measurement data during this study domain and period. In another study period, where the abundance of long-term (> 1yr) flux observations is available, future studies could suggest improved parameters for the Korean Peninsula.

## Conclusions

Over a study domain, the South Korean Peninsula, we conducted numerical simulations of atmospheric CO_2_, using a coupled WRF-VPRM model. The model’s performance was evaluated by comparing with observations acquired from ground CO_2_ monitoring sites, a TCCON spectrometer, a CO_2_ flux site, and NASA DC-8 aircraft observations during the KORUS-AQ 2016 field campaign. WRF-VPRM successfully captured the spatiotemporal variation of CO_2_ concentrations over the South Korean Peninsula, strongly associated with the temporal variations of PBL and the spatial distribution of emission sources and biosphere.

The simulated results were comparable with the ground CO_2_ concentrations observed at two CO_2_ monitoring sites, GO (MB ~ -0.4 ppm) and UL (MB ~ -0.7 ppm), and with the CO_2_ column concentration from the TCCON spectrometer at AN (MB ~ -0.3 ppm). Beside the monitoring sites, we also compared simulations with tower observations at BO. The model significantly underestimated the concentration at 60 m at night and the vertical gradient of CO_2_ from tower observations (BO). This large uncertainty was likely due to the unresolved local emission sources in the model.

Our model successfully captured the diurnal variation of CO_2_ concentrations at 300m, and the simulated results underestimated CO_2_ by approximately 3.9 ppm (RMSE = 9.6) and 0.5 ppm (RMSE = 18.8) in day- and nighttime. At 60 m, on the other hand, the model failed to capture the amplitude of temporal variations and significantly underestimated nighttime concentrations by 10.3 ppm (RMSE = 14.3).

Besides the monitoring sites, the model’s performance was also evaluated at an inland tower site at BO. WRF-VPRM underestimated the CO_2_ concentration by ~3 ppm at 300 m and ~9 ppm at 60 m. The largest bias occurred in nighttime at 60 m. During the study period, there was a nighttime inversion layer, the height of which was located between 60 m and 300 m. Considering the model’s successful simulated results of the nighttime stable (nocturnal) boundary layer, the main culprit of the significant underestimation likely resides with the fact that the relatively coarser emission data FFDAS could not fully resolve the local sources around BO (Sect. 3.2).

At higher altitudes, the simulated results were evaluated with the NASA DC-8 aircraft observations, separated into four flight groups: SMA, the West Sea, and the J- and B-route. The model captured variations in CO_2_ concentrations along the DC-8 flight tracks, depending on horizontal and vertical locations. However, it failed to capture large concentrations spikes appearing in the PBL, especially over the SMA where Seoul urban emissions are dominant, and over the Taeahn Peninsula, where power plants and industrial complexes are densely located. Overall, the western part of Korea contains the dominant emission sources in the study domain.

The influence of long-range transport of CO_2_ from China was also investigated on May 25, when the DC-8 aircraft flew along the “wall” flight track over the West Sea. The model captured the time series of CO_2_ concentrations. To investigate the effect of plume transported from China, we conducted additional simulations in which only Chinese emissions were established. Unexpectedly, the magnitude of concentrations transported from China was very low compared to the background CO_2_ concentration. Therefore, we were not able to quantify any significant CO_2_ advection from China from this particular case.

In addition to the evaluation of CO_2_ concentrations, we also compared the simulated NEE with measured CO_2_ fluxes at a flux tower to evaluate the modeling performance for the CO_2_ exchange between the biosphere and the atmosphere. Although the model captured the temporal variability in the fluxes: negative and positive values during day- and night-time, respectively, it showed a significant offset compared with the observed fluxes. VPRM parameters are variable season by season and region by region, so they should be optimized at each vegetation type. Further optimization of VPRM parameters could lead to better performance, as suggested in previous studies, but was not possible for this study due to the lack of available sufficient flux data at various vegetation types over a long time period (> 1 yr).

The output of this modeling work can be used to validate remote sensing instruments, such as GOSAT and OCO-2. The results are also useful to support environmental mitigation policy of the Ministry of Environment, especially for the fossil-fuel carbon emission controls, and/or biospheric management in Korean Government.

## Supporting information

S1 Table Statistics for meteorology for 11 sensitivity tests for d01 from May 12 to June. (DOCX)

S1 Fig Spatial distribution of emissions in d02. The color code indicates the emission range at each grid pixel (unit: mole km^-2^ hr^-1^).
(TIF)

S2 Fig Diurnal variation of CO_2_ concentrations and meteorology observed at the CO_2_ flux tower site BO at 60 m and 300 m a.g.l from 2016 to 2019 only for spring. (TIF)

S3 Fig The same as Fig 6, but for all flight days from May 3 to May 25. (TIF)

S4 Fig The same as S3 Fig, but for the flight days from May 26 to June 10. (TIF)
