# Supplementary material for: Numerical simulation of atmospheric CO2 concentration and flux over the Korean Peninsula using WRF-VPRM model during Korus-AQ 2016 campaign
Source: PLoS One. 2020 Jan 24;15(1):e0228106. doi: 10.1371/journal.pone.0228106 (PMC6980530; doi:10.1371/journal.pone.0228106)
Supplement: S1 Table — (DOCX) [file pone.0228106.s001.docx]

S1 Table. Statistics for meteorology for 11 sensitivity tests for d01 from May 12 to June 10.

| Option names |  | RMSE | MB | IOA | R | N |
| --- | --- | --- | --- | --- | --- | --- |
| YSU | T2m | 2.8 | -1.7 | 0.90 | 0.88 | 32745 |
|  | WS | 1.5 | 0.8 | 0.70 | 0.56 | 32745 |
|  | WD | 3.5 | -2.9 | 0.48 | 0.32 | 32745 |
| YSU_pblmix | T2m | 2.7 | -1.7 | 0.90 | 0.88 | 32745 |
|  | WS | 1.5 | 0.8 | 0.70 | 0.56 | 32745 |
|  | WD | 3.5 | -2.9 | 0.48 | 0.32 | 32745 |
| YSU_topowind | T2m | 2.7 | -1.7 | 0.90 | 0.88 | 32745 |
|  | WS | 1.5 | **0.4** | 0.66 | 0.46 | 32745 |
|  | WD | 3.5 | -2.9 | 0.48 | 0.32 | 32745 |
| **YSU_topowind.pblmix** | T2m | 2.7 | **-1.6** | 0.90 | 0.88 | 32745 |
|  | WS | 1.5 | **0.4** | 0.66 | 0.46 | 32745 |
|  | WD | 3.5 | -2.9 | 0.48 | 0.32 | 32745 |
| MYJ | T2m | 2.6 | -1.4 | 0.90 | 0.87 | 32745 |
|  | WS | 1.7 | 1.0 | 0.66 | 0.56 | 32745 |
|  | WD | 3.5 | -2.9 | 0.48 | 0.32 | 32745 |
| MYNN2 | T2m | 3.3 | -2.4 | 0.87 | 0.87 | 32745 |
|  | WS | 1.5 | 0.7 | 0.72 | 0.59 | 32745 |
|  | WD | 3.5 | -2.9 | 0.48 | 0.32 | 32745 |
| MYNN3 | T2m | 3.4 | -2.6 | 0.85 | 0.86 | 32745 |
|  | WS | 1.5 | 0.7 | 0.72 | 0.58 | 32745 |
|  | WD | 3.5 | -2.9 | 0.48 | 0.31 | 32745 |
| ACM2 | T2m | 3.0 | -1.5 | 0.91 | 0.88 | 32745 |
|  | WS | 1.5 | 0.7 | 0.71 | 0.57 | 32745 |
|  | WD | 3.5 | -2.9 | 0.49 | 0.33 | 32745 |
| BOULAC | T2m | 2.7 | -1.5 | 0.91 | 0.87 | 32745 |
|  | WS | 1.5 | 0.7 | 0.69 | 0.54 | 32745 |
|  | WD | 3.5 | -2.9 | 0.48 | 0.32 | 32745 |
| TEMF | T2m | 3.1 | -0.4 | 0.89 | 0.81 | 32745 |
|  | WS | 1.5 | 0.8 | 0.69 | 0.54 | 32745 |
|  | WD | 3.5 | -2.9 | 0.48 | 0.31 | 32745 |
| Shing-Hong | T2m | 2.8 | -1.8 | 0.90 | 0.88 | 32745 |
|  | WS | 1.5 | 0.8 | 0.70 | 0.56 | 32745 |
|  | WD | 3.5 | -2.9 | 0.48 | 0.32 | 32745 |
|  | | | | | | |
