# Supplementary figures and images for: Numerical simulation of atmospheric CO2 concentration and flux over the Korean Peninsula using WRF-VPRM model during Korus-AQ 2016 campaign
Source: PLoS One. 2020 Jan 24;15(1):e0228106. doi: 10.1371/journal.pone.0228106 (PMC6980530; doi:10.1371/journal.pone.0228106)

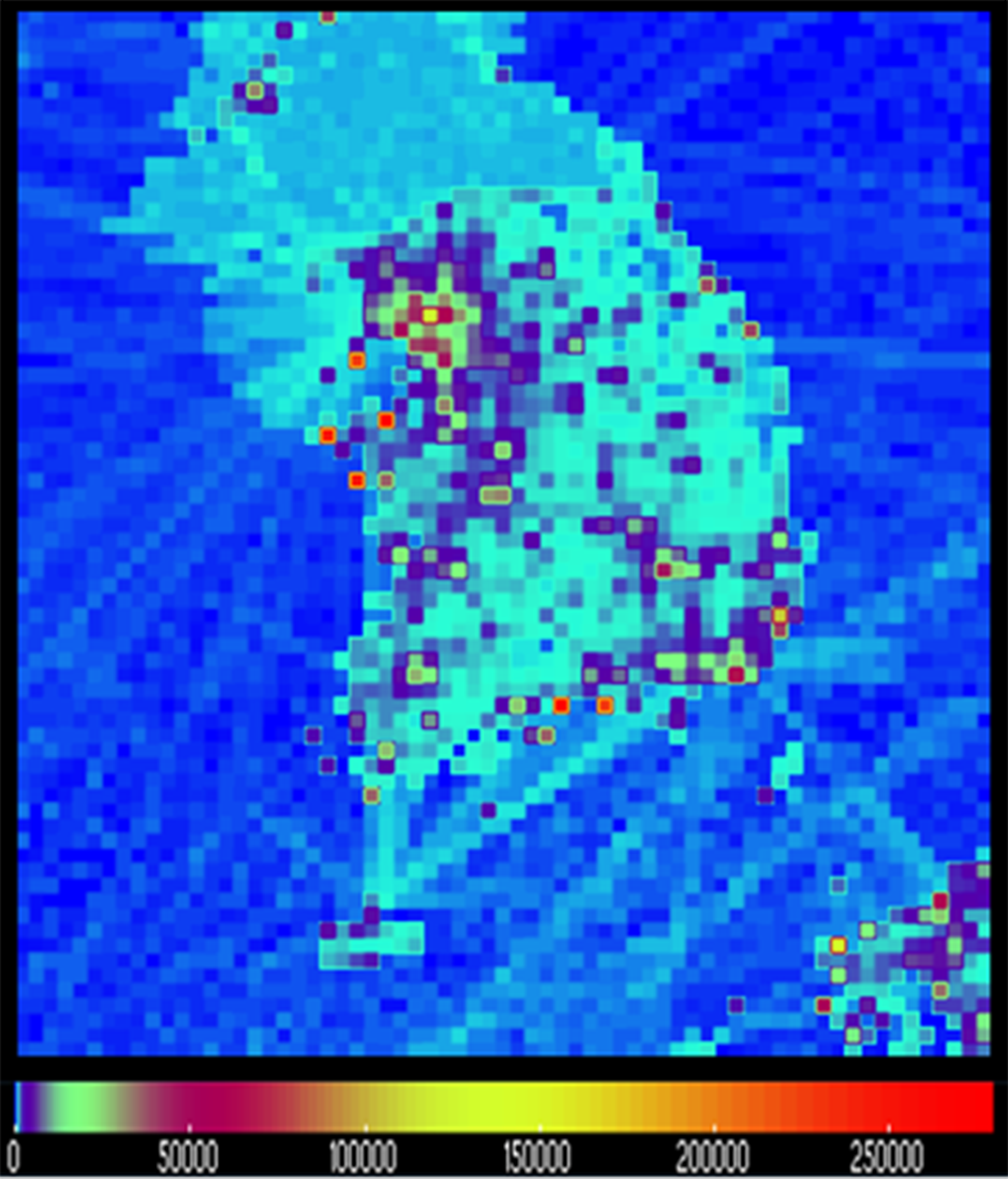

Supplement: S1 Fig — The color code indicates the emission range at each grid pixel (unit: mole km-2 hr-1). (TIF) [file pone.0228106.s002.tif]

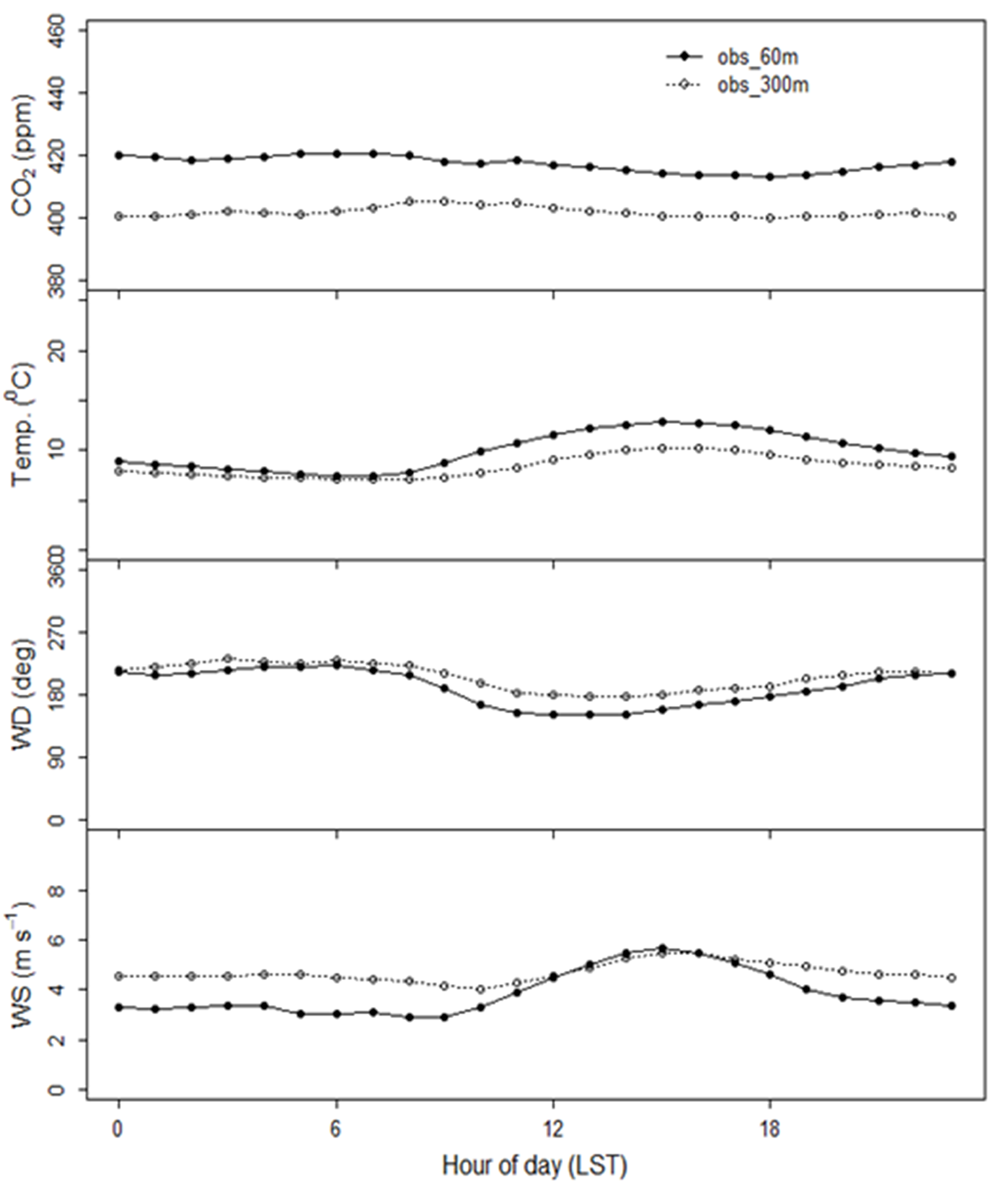

Supplement: S2 Fig — (TIF) [file pone.0228106.s003.tif]

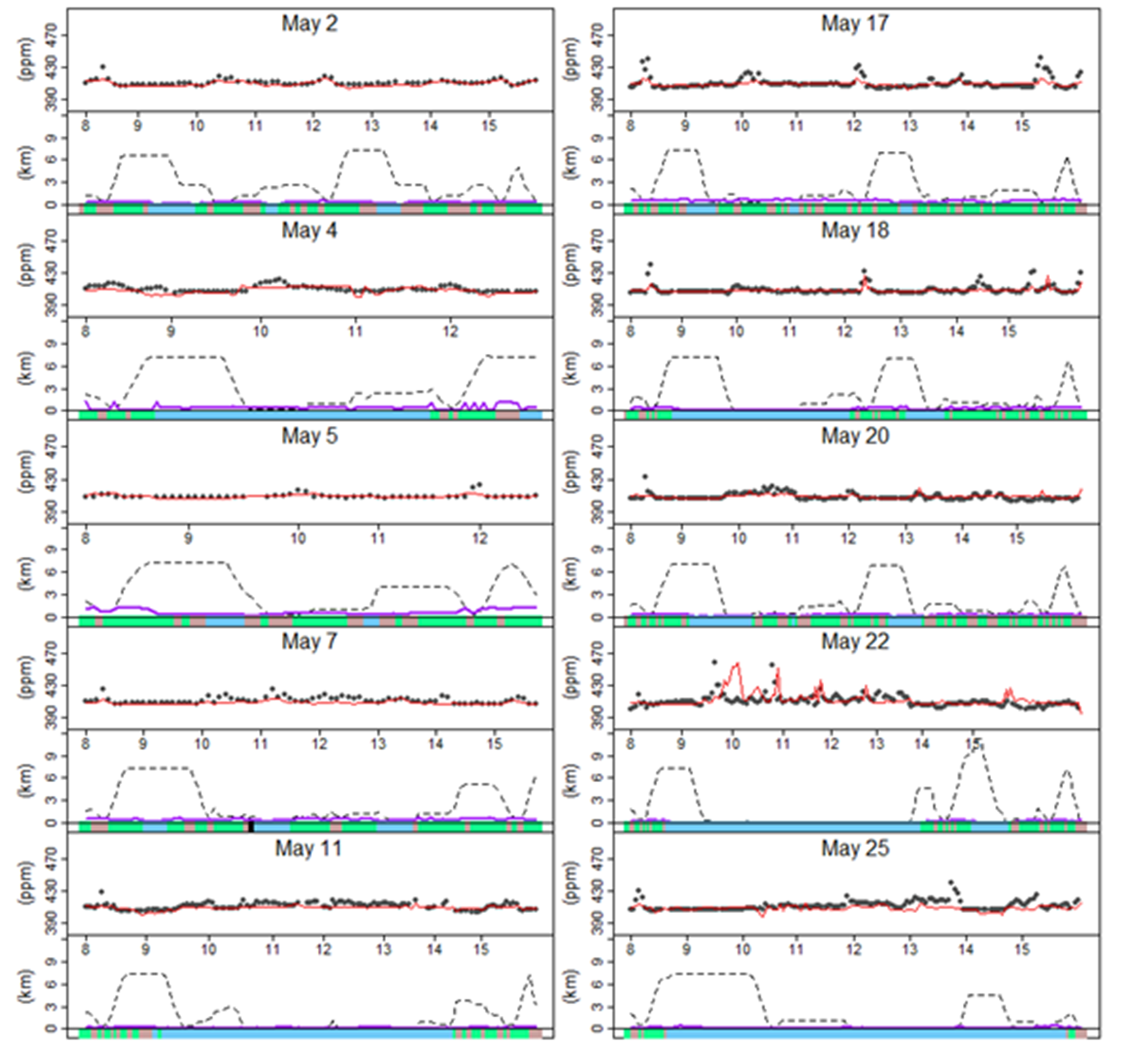

Supplement: S3 Fig — (TIF) [file pone.0228106.s004.tif]

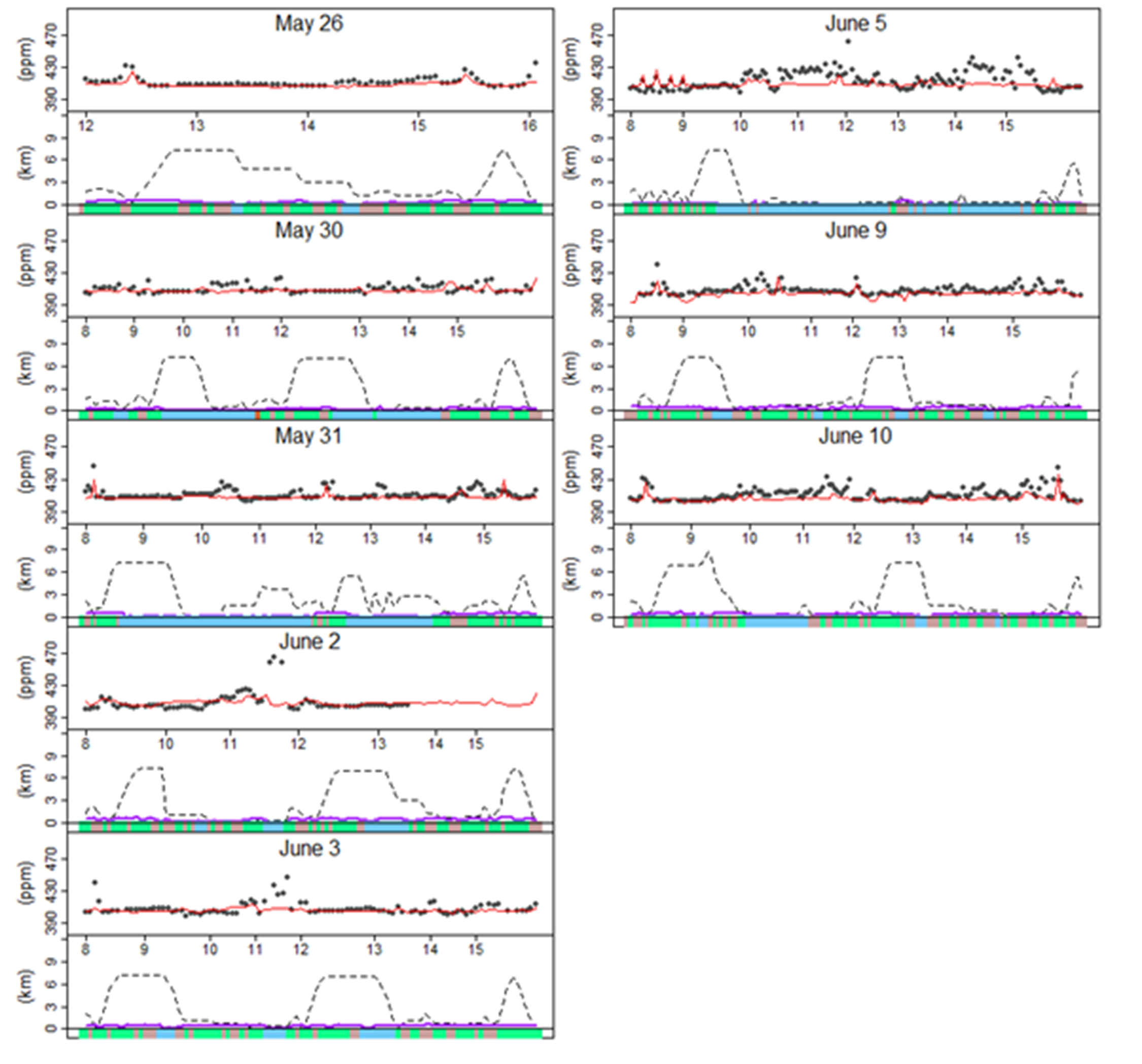

Supplement: S4 Fig — (TIF) [file pone.0228106.s005.tif]
